# Temporal trends of catheter ablation procedures in patients with atrial fibrillation and atrial flutter: A nationwide cohort study

**DOI:** 10.1016/j.ijcha.2024.101541

**Published:** 2024-10-23

**Authors:** Antti Lappalainen, Juha E.K. Hartikainen, Konsta Teppo, Olli Halminen, Aapo L. Aro, Rasmus Siponen, Janne Virrankorpi, Annukka Marjamaa, Birgitta Salmela, Jukka Putaala, Pirjo Mustonen, Miika Linna, Jari Haukka, K.E. Juhani Airaksinen, Mika Lehto

**Affiliations:** a Heart Centre, Kuopio University Hospital and University of Eastern Finland, Kuopio, Finland; b Heart Centre, Turku University Hospital and University of Turku, Turku, Finland; c Department of Industrial Engineering and Management, Aalto University, Espoo, Finland; d University of Eastern Finland, Kuopio, Finland; e Faculty of Medicine, University of Helsinki, Helsinki, Finland; f Heart and Lung Center, Helsinki University Hospital and University of Helsinki, Helsinki, Finland; g Heart Center, Department of Internal Medicine, Päijät-Häme Central Hospital, Lahti, Finland; h Department of Neurology, Helsinki University Hospital and University of Helsinki, Helsinki, Finland; i Department of Health and Social Management, University of Eastern Finland, Kuopio, Finland; j Department of Internal Medicine, Jorvi Hospital, HUS Helsinki University Hospital and University of Helsinki, Helsinki, Finland

**Keywords:** Atrial fibrillation, Atrial flutter, Catheter ablation

## Abstract

**Background:**

Catheter ablation is a well-established treatment to prevent atrial fibrillation (AF) and atrial flutter (AFL) recurrences and to relieve symptoms, whereas pacemaker implantation and atrioventricular node (AVN) ablation is used for rate control when medical therapy fails.

**Aims:**

We investigated temporal trends and patient characteristics in catheter ablation procedures for AF, AFL and AVN in Finland between 2012–2018.

**Methods:**

Finnish AntiCoagulation in Atrial Fibrillation (FinACAF) is a registry-based study including all patients with AF or AFL in Finland between 2012–2018.

**Results:**

The number of patients with AF or AFL diagnosis in Finland increased from 185 057 to 243 802 between 2012–2018 and a total of 8954 first-time catheter ablation procedures were performed. Of them, 4909 (54.8 %) were AF ablations, 2731 (30.5 %) AFL ablations and 1314 (14.7 %) AVN ablations. The procedural numbers increased from 457/year to 934/year for AF, from 223/year to 553/year for AFL and from 114/year to 238/year for AVN. Altogether, 0.65% of all patients with diagnosed AF or AFL underwent AF, AFL or AVN ablation in 2018. The mean age of the patients increased in all ablation groups. Patients undergoing AF and AFL ablations were predominantly men (69.7 % and 74.6 % respectively) whereas patients undergoing AVN ablation were more often women (56.9%).

**Conclusions:**

The use of catheter ablation more than doubled during 2012–2018 and the increase was particularly seen in the elderly patients. Nevertheless, only a minority of AF and AFL patients were treated with catheter ablations.

## 1 Introduction

Atrial fibrillation (AF) is the most common sustained cardiac rhythm disorder with increasing prevalence due to ageing population. [1], [2], [3], [4] AF is associated with substantial morbidity and mortality and it causes a significant burden to the healthcare systems. Thus, significant research efforts and resources have been allocated to find more effective ways to identify AF and treat it. AF and atrial flutter (AFL) often coexist, and a considerable proportion of patients with AFL will later develop AF, even though AFL can also exist as an isolated arrhythmia. [5].

Catheter ablation is a well-established and safe treatment to prevent AF and AFL recurrences and it has been shown to be a superior to antiarrhythmic drugs (AAD) for maintaining sinus rhythm and to decrease AF and AFL related symptoms. [5], [6] Current guidelines recommend catheter ablation in symptomatic patients with paroxysmal or persistent AF who are refractory or intolerant to AADs. Catheter ablation may also be considered as a first-line treatment in selected patients according to the patient’s preferences and particularly in patients with heart failure and patients with reduced left ventricular ejection fraction due to AF induced cardiomyopathy. [5], [7] Pacemaker implantation and atrioventricular node (AVN) ablation is a treatment strategy for achieving sufficient rate control in AF or AFL when other treatment options have failed. [5], [8], [9].

In the 2012 Focused update of the European Society of Cardiology AF Guidelines, catheter ablation of symptomatic AF recurrences after failed AAD therapy was upgraded to class I recommendation from previous class IIb. [10] The upgrade followed the American recommendations at the time and the emphasis on catheter ablation treatment for AF has increased ever since in the upcoming guidelines. In recent years, several studies have suggested outcome benefits of an early and active rhythm control strategy. [11], [12] However, comprehensive data on the real-life implementation of the clinical practise guidelines and temporal trends in the use of catheter ablation in patients with AF and AFL are sparce. Particularly, information on the trends in the clinical characteristics of the patients scheduled for these procedures remains limited. Therefore, we conducted a nationwide retrospective cohort study covering all patients with AF or AFL and explored the trends in procedural volumes and characteristics of patients who were treated with invasive rhythm and rate control strategies in Finland between 2012 and 2018.

## 2 Methods

### 2.1 Study population

The Finnish AntiCoagulation in Atrial Fibrillation (FinACAF) Study (ClinicalTrials Identifier: NCT04645537; ENCePP Identifier: EUPAS29845) is a nationwide retrospective cohort study including all patients documented with AF and AFL in Finland from 2004 to 2018 encompassing a total of 411 387 patients. Patients were identified using all national healthcare registers, including hospitalizations and outpatient specialist visits (HILMO), primary healthcare (AvoHILMO from 2012 onwards), and the National Reimbursement Register maintained by the Social Insurance Institute (KELA). The cohort inclusion criterion was an International Classification of Diseases, Tenth Revision (ICD-10) diagnosis code of I48, encompassing AF and AFL. The Nordic Classification of Surgical Procedures (NCSP) codes identifying AF, AFL and AVN catheter ablations were available in Finland from the registries since 2011. In this sub-study we excluded patients under 18 years old at the time of cohort entry and searched all patients with procedural codes of AF catheter ablation (TFP46 including all methods for AF ablation), typical AFL catheter ablation (TFP44), atypical AFL catheter ablation (TFP45) and AVN catheter ablation (TFP47) (Appendix 1). Because these procedural codes were introduced in 2011, we started the evaluation of procedural trends from 2012 and ignored the year 2011 as a transition year. We defined baseline characteristics of the patients at the time of the first catheter ablation procedure. We also identified those who had AF or AFL redo-ablations after 30 days blanking period from the first ablation and compared the characteristics of these patients to those undergoing only single ablation procedure. We calculated the proportion of patients who underwent catheter ablations within two and five years of cohort entry and time from the first to the second catheter ablation. Moreover, we identified the proportion of patients who had purchased beta-blockers or AADs (flecainide, amiodarone, dronedarone or sotalol) within one year before the first catheter ablation.

### 2.2 Study ethics

The study protocol was approved by the Ethics Committee of the Medical Faculty of Helsinki University, Helsinki, Finland (nr. 15/2017) and received research permission from the Helsinki University Hospital (HUS/46/2018). Respective permissions were obtained from the Finnish register holders (KELA 138/522/2018; THL 2101/5.05.00/2018; Population Register Centre VRK/1291/2019–3; Statistics Finland TK-53–1713–18 / u1281; and Tax Register VH/874/07.01.03/2019). All Finnish inhabitants have a unique national identification number, and the patients’ individual data from Finnish nationwide population registers and regional laboratory databases were linked together, using this national identification code. The research group received individualized, but pseudonymized and unidentifiable data which ensures full data protection of the patients. The study was conducted without any direct patient involvement or contact during any phase of the study. Therefore, no patient consent was needed according to the Finnish legislation. [1], [2] The study conforms to the Declaration of Helsinki as revised in 2013.

### 2.3 Statistical analyses

Differences in categorical variables were compared using Chi-square test and continuous variables were compared using students *t*-test and one-way analysis of variance. Statistical analyses were conducted using IBM SPSS Statistics software version 29.0 (SPSS, Inc., Chicago, Illinois, USA).

## 3 Results

### 3.1 Study population

The total amount of patients with prevalent AF and AFL in the Finnish adult population increased from 185 057 patients in 2012 to 243 802 patients in 2018 and a total of 8954 first-time catheter ablation procedures on 7915 patients were performed during 2012–2018. Of them, 4909 (54.8 %) were AF ablations, 2731 (30.5 %) AFL ablations and 1314 (14.7 %) AVN ablations (Table 1, Table 2, Table 3). A great majority of patients undergoing catheter ablation for AF (69.7 %) and AFL (74.6 %) were men, whereas most of the patients undergoing AVN ablation (56.9 %) were women. Mean age at the time of the first procedure were 59.1 years in AF, 62.3 years in AFL and 73.0 years in AVN ablation groups (Table 1, Table 2, Table 3). Women were older than men across all ablation types: AF (62.4 vs. 57.6 years), AFL (64.5 vs. 61.5 years), and AVN (76.1 vs. 70.0 years) (p < 0.001 for all).

**Table 1:** First-time catheter ablation procedures of atrial fibrillation.

	**2012**	**2013**	**2014**	**2015**	**2016**	**2017**	**2018**	**Total**	**p-value**
**n**	457	570	594	691	788	875	934	4909	
**Age**	58.0 (8.8)	57.2 (9.7)	58.3 (9.9)	57.8 (10.3)	59.6 (9.7)	60.5 (9.6)	60.5 (10.3)	59.1 (9.9)	**< 0.001**
Range	29–78	28–76	21–79	24–81	25–84	25–87	24–86	21–87	
**Age groups**	**n (%)**								
18–30 years	1 (0.2)	3 (0.5)	8 (1.3)	8 (1.2)	5 (0.6)	5 (0.6)	9 (1.0)	39 (0.8)	0.203
30–39 years	15 (3.3)	32 (5.6)	27 (4.5)	32 (4.6)	27 (3.4)	28 (3.2)	34 (3.6)	195 (4.0)	0.227
40–49 years	66 (14.4)	87 (15.3)	73 (12.3)	112 (16.2)	92 (11.7)	70 (8.0)	93 (10.0)	593 (12.1)	**< 0.001**
50–59 years	157 (34.4)	187 (32.8)	202 (34.0)	208 (30.1)	252 (32.0)	283 (32.3)	277 (29.7)	1566 (31.9)	0.427
60–69 years	191 (41.8)	231 (40.5)	231 (38.9)	274 (39.7)	314 (39.8)	351 (40.1)	342 (36.6)	1934 (39.4)	0.574
70–79 years	27 (5.9)	30 (5.3)	53 (8.9)	56 (8.1)	95 (12.1)	135 (15.4)	175 (18.7)	571 (11.6)	**< 0.001**
80 years and older	0 (0)	0 (0)	0 (0)	1 (0.1)	3 (0.3)	3 (0.3)	4 (0.4)	11 (0.2)	0.321
**Female**	119 (26.0)	161 (28.2)	181 (30.5)	198 (28.7)	250 (31.7)	283 (32.3)	294 (31.5)	1486 (30.3)	0.170
**Comorbidities**									
Hypertension	287 (62.8)	346 (60.7)	367 (61.8)	415 (60.1)	464 (58.9)	524 (59.9)	552 (59.1)	2955 (60.2)	0.802
Diabetes	25 (5.5)	37 (6.5)	44 (7.4)	49 (7.1)	59 (7.5)	78 (8.9)	74 (7.9)	366 (7.5)	0.369
Hyperlipidaemia	118 (25.8)	148 (26.0)	170 (28.6)	197 (28.5)	227 (28.8)	258 (29.5)	286 (30.6)	1404 (28.6)	0.436
Heart failure	28 (6.1)	31 (5.4)	39 (6.6)	37 (5.4)	51 (6.5)	42 (4.8)	70 (7.5)	298 (6.1)	0.294
Coronary artery disease	80 (17.5)	104 (18.2)	93 (15.7)	88 (12.7)	121 (15.4)	103 (11.8)	138 (14.8)	727 (14.8)	**0.008**
Ischemic stroke or TIA	41 (9.0)	31 (5.4)	41 (6.9)	55 (8.0)	75 (9.5)	90 (10.3)	77 (8.2)	410 (8.4)	**0.030**
Abnormal renal function	0 (0)	2 (0.4)	3 (0.5)	3 (0.4)	6 (0.8)	8 (0.9)	11 (1.2)	33 (0.7)	0.160
Abnormal liver function	1 (0.2)	2 (0.4)	0 (0)	0 (0)	3 (0.4)	2 (0.2)	2 (0.2)	10 (0.2)	0.612
Previous bleedings	36 (7.9)	36 (6.3)	53 (8.9)	50 (7.2)	51 (6.5)	59 (6.7)	80 (8.6)	365 (7.4)	0.367
Alcohol abuse	10 (2.2)	6 (1.1)	9 (1.5)	17 (2.5)	18 (2.3)	17 (1.9)	25 (2.7)	102 (2.1)	0.392
CHA_2_DS_2_-VASc	1.6 (1.2)	1.6 (1.3)	1.7 (1.3)	1.6 (1.3)	1.8 (1.4)	1.8 (1.5)	1.8 (1.5)	1.7 (1.4)	**< 0.001**
CHA_2_DS_2_-VA	1.3 (1.1)	1.3 (1.1)	1.4 (1.1)	1.3 (1.1)	1.5 (1.3)	1.5 (1.3)	1.5 (1.3)	1.4 (1.2)	**0.002**

Values denote n (%) or means with standard deviations. Abbreviations: CHA_2_DS_2_-VASc score, congestive heart failure (1 point), hypertension (1 point), age ≥ 75 years (2 points), diabetes (1 point), history of stroke or TIA (2 points), vascular disease (1 point), age 65–74 years (1 point), sex category (female) (1 point). TIA, transient ischaemic attack.

**Table 2:** First-time catheter ablation procedures of atrial flutter.

	**2012**	**2013**	**2014**	**2015**	**2016**	**2017**	**2018**	**Total**	**p-value**
**n**	223	232	315	380	481	547	553	2731	
**Age**	60.4 (10.5)	61.3 (11.0)	60.4 (11.1)	61.1 (11.3)	62.9 (10.5)	62.8 (11.2)	64.2 (10.7)	62.3 (11.0)	**< 0.001**
**Age, range**	19–85	23–84	23–87	20–86	27–88	22–98	23–86	19–98	
**Age groups**									
18–30 years	2 (0.9)	3 (1.3)	6 (1.9)	4 (1.1)	2 (0.4)	4 (0.7)	4 (0.7)	25 (0.9)	0.470
30–39 years	9 (4.0)	9 (3.9)	10 (3.2)	17 (4.5)	11 (2.3)	23 (4.2)	13 (2.4)	92 (3.4)	0.358
40–49 years	22 (9.9)	17 (7.3)	28 (8.9)	45 (11.8)	41 (8.5)	32 (5.9)	35 (6.3)	220 (8.1)	**0.022**
50–59 years	67 (30.0)	61 (26.3)	96 (30.5)	80 (21.1)	122 (25.4)	141 (25.8)	120 (21.7)	687 (25.2)	**0.022**
60–69 years	86 (38.6)	97 (41.8)	124 (39.4)	150 (39.5)	173 (36.0)	203 (37.1)	195 (35.3)	1028 (37.6)	0.581
70–79 years	33 (14.8)	40 (17.2)	44 (14.0)	79 (20.8)	118 (24.5)	122 (22.3)	164 (29.7)	600 (22.0)	**< 0.001**
80 years and older	4 (1.8)	5 (2.2)	7 (2.2)	5 (1.3)	14 (2.9)	22 (4.0)	22 (4.0)	79 (2.9)	0.121
Female	40 (17.9)	53 (22.8)	69 (21.9)	97 (25.5)	112 (23.3)	163 (29.8)	160 (28.9)	694 (25.4)	**0.003**
**Comorbidities**									
Hypertension	148 (66.4)	148 (63.8)	184 (58.4)	231 (60.6)	312 (64.9)	342 (62.5)	362 (65.5)	1727 (63.2)	0.326
Diabetes	29 (13.0)	31 (13.4)	46 (14.6)	50 (13.1)	88 (18.3)	76 (13.9)	97 (17.5)	417 (15.3)	0.154
Hyperlipidaemia	82 (36.8)	81 (34.9)	113 (35.9)	134 (35.2)	186 (38.7)	208 (38.0)	230 (41.6)	1034 (37.9)	0.423
Heart failure	35 (15.7)	30 (12.9)	51 (16.2)	61 (16.0)	72 (15.0)	92 (16.8)	107 (19.3)	448 (16.4)	0.374
Coronary artery disease	53 (23.8)	55 (23.7)	61 (19.4)	67 (17.6)	95 (19.8)	103 (18.8)	109 (19.7)	543 (19.9)	0.435
Ischemic stroke or TIA	20 (9.0)	14 (6.0)	22 (7.0)	25 (6.6)	39 (8.1)	43 (7.9)	50 (9.0)	213 (7.8)	0.709
Abnormal renal function	1 (0.4)	3 (1.3)	6 (1.9)	11 (2.9)	15 (3.1)	14 (2.6)	11 (2.0)	61 (2.2)	0.298
Abnormal liver function	0 (0)	1 (0.4)	0 (0)	1 (0.3)	3 (0.6)	5 (0.9)	1 (0.2)	11 (0.4)	0.303
Previous bleedings	19 (8.5)	17 (7.3)	24 (7.6)	33 (8.7)	55 (11.4)	69 (12.6)	65 (11.8)	282 (10.3)	0.072
Alcohol abuse	7 (3.1)	4 (1.7)	6 (1.9)	18 (4.7)	24 (5.0)	20 (3.7)	21 (3.8)	100 (3.7)	0.171
CHA_2_DS_2_-VASc	2.0 (1.5)	2.0 (1.5)	1.9 (1.5)	2.0 (1.5)	2.2 (1.6)	2.2 (1.6)	2.4 (1.7)	2.2 (1.6)	**< 0.001**
CHA_2_DS_2_-VA	1.8 (1.4)	1.8 (1.4)	1.7 (1.4)	1.8 (1.5)	2.0 (1.5)	1.9 (1.5)	2.1 (1.6)	1.9 (1.5)	**< 0.001**

Values denote n (%) or means with standard deviations. Abbreviations: CHA_2_DS_2_-VASc score, congestive heart failure (1 point), hypertension (1 point), age ≥ 75 years (2 points), diabetes (1 point), history of stroke or TIA (2 points), vascular disease (1 point), age 65–74 years (1 point), sex category (female) (1 point). TIA, transient ischaemic attack.

.

**Table 3:** First-time catheter ablation procedures of atrioventricular node.

	**2012**	**2013**	**2014**	**2015**	**2016**	**2017**	**2018**	**Total**	**p-value**
**n**	114	122	164	197	223	256	238	1314	
**Age**	70.6 (10.4)	72.2 (10.2)	71.9 (10.5)	72.3 (9.9)	73.8 (9.2)	74.4 (9.2)	73.6 (10.4)	73.0 (9.9)	**0.007**
Range	36–86	48–93	34–93	26–92	32–95	41–96	27–94	26–96	
**Age groups**	**n (%)**								
18–30 years	0 (0)	0 (0)	0 (0)	1 (0.5)	0 (0)	0 (0)	1 (0.4)	2 (0.2)	0.663
30–39 years	1 (0.9)	0 (0)	2 (1.2)	1 (0.5)	1 (0.4)	0 (0)	0(0)	5 (0.4)	0.396
40–49 years	2 (1.8)	3 (2.5)	0 (0)	2 (1.0)	2 (0.9)	1 (0.4)	6 (2.5)	16 (1.2)	0.177
50–59 years	13 (11.4)	12 (9.8)	18 (11.0)	15 (7.6)	12 (5.4)	19 (7.4)	20 (8.4)	109 (8.3)	0.396
60–69 years	32 (28.1)	34 (27.9)	45 (27.4)	49 (24.9)	49 (22.0)	52 (20.3)	37 (15.5)	298 (22.7)	**0.027**
70–79 years	39 (34.2)	41 (33.6)	60 (30.6)	77 (39.1)	99 (44.4)	106 (41.4)	93 (39.1)	515 (39.2)	0.389
80 years and older	27 (23.7)	32 (26.2)	39 (23.8)	52 (26.4)	60 (26.9)	78 (30.5)	81 (34.0)	369 (28.1)	0.229
**Female**	73 (64.0)	68 (55.7)	97 (59.1)	109 (55.3)	125 (56.1)	143 (55.9)	133 (55.9)	748 (56.9)	0.777
**Comorbidities**									
Hypertension	94 (82.5)	104 (85.2)	141 (86.0)	165 (83.8)	184 (82.5)	222 (86.7)	196 (82.4)	1106 (84.2)	0.798
Diabetes	31 (27.2)	31 (25.4)	54 (32.9)	59 (29.9)	55 (24.7)	69 (27.0)	67 (28.2)	366 (27.9)	0.655
Hyperlipidaemia	56 (49.1)	65 (53.3)	91 (55.5)	102 (51.8)	132 (59.2)	142 (55.5)	126 (52.9)	714 (54.3)	0.629
Heart failure	69 (60.5)	69 (56.6)	96 (58.5)	121 (61.4)	139 (62.3)	154 (60.2)	155 (65.1)	803 (61.1)	0.757
Coronary artery disease	46 (40.4)	58 (47.5)	73 (44.5)	78 (39.6)	100 (44.8)	105 (41.0)	101 (42.4)	561 (42.7)	0.790
Ischemic stroke or TIA	17 (14.9)	19 (15.6)	34 (20.7)	47 (23.9)	41 (18.4)	42 (16.4)	38 (16.0)	238 (18.1)	0.260
Abnormal renal function	6 (5.3)	9 (7.4)	14 (8.5)	15 (7.6)	12 (5.4)	27 (10.5)	19 (8.0)	102 (7.8)	0.456
Abnormal liver function	1 (0.9)	1 (0.8)	1 (0.6)	0 (0)	1 (0.4)	1 (0.4)	0 (0)	5 (0.4)	0.773
Previous bleedings	12 (10.5)	15 (12.3)	29 (17.7)	36 (18.3)	39 (17.5)	54 (21.1)	53 (22.3)	238 (18.1)	0.072
Alcohol abuse	4 (3.5)	2 (1.6)	0 (0)	6 (3.0)	5 (2.2)	12 (4.7)	7 (2.9)	36 (2.7)	0.153
CHA_2_DS_2_-VASc	4.3 (1.6)	4.3 (1.9)	4.5 (1.9)	4.5 (1.8)	4.5 (1.7)	4.5 (1.8)	4.5 (1.7)	4.4 (1.7)	0.527
CHA_2_DS_2_ −VA	3.6 (1.5)	3.7 (1.7)	3.9 (1.7)	3.9 (1.6)	3.9 (1.6)	3.9 (1.6)	3.9 (1.5)	3.9 (1.6)	0.641

Values denote n (%) or means with standard deviations. Abbreviations: CHA_2_DS_2_-VASc score, congestive heart failure (1 point), hypertension (1 point), age ≥ 75 years (2 points), diabetes (1 point), history of stroke or TIA (2 points), vascular disease (1 point), age 65–74 years (1 point), sex category (female) (1 point). TIA, transient ischaemic attack.

The most common comorbidity among the catheter ablation patients was hypertension (60.2 %, 63.2 % and 84.2 % in patients with AF, AFL and AVN ablations, respectively) (Table 1, Table 2, Table 3). The AVN ablation group had the highest prevalence of comorbidities as in addition to hypertension, 27.9 % presented with diabetes, 54.3 % with hyperlipidaemia, 61.1 % with heart failure, 42.7 % with coronary artery disease and 18.1 % with a history of ischaemic stroke or TIA.

### 3.2 Temporal trends in ablations

**Fig. 1:**
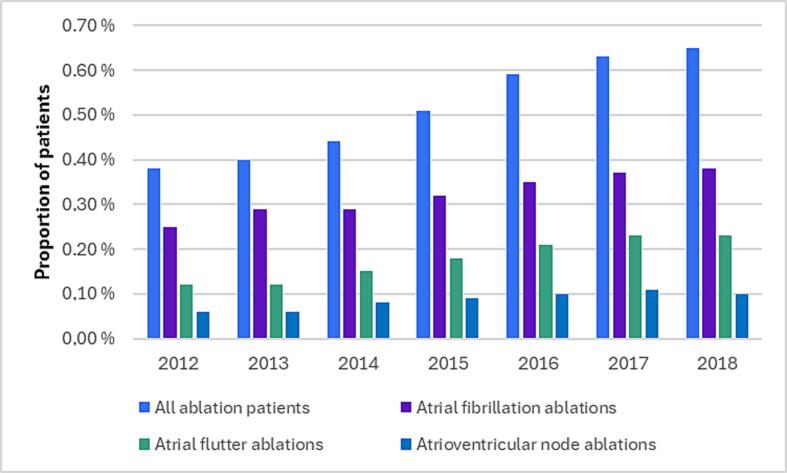
Proportion of patients with first-time catheter ablations in total AF and AFL population by year.

The annual numbers of first-time catheter ablation procedures increased from 457/year to 934/year for AF, from 223/year to 553/year for AFL and from 114/year to 238/year for AVN. In 2012 0.38 % of all patients with diagnosed AF or AFL in Finland underwent either AF, AFL or AVN ablation and by 2018 the proportion almost doubled to 0.65 % (Fig. 1, Appendix 2).

**Fig. 2:**
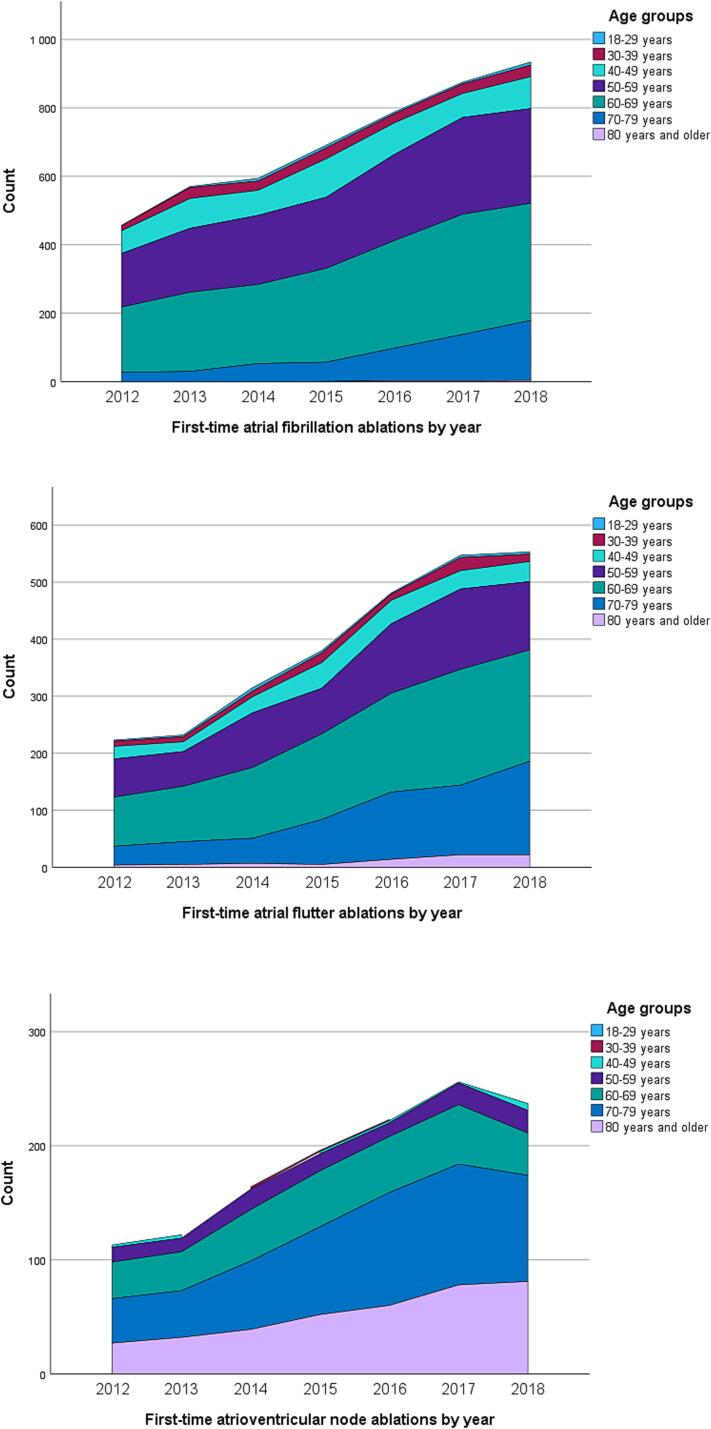
First-time catheter ablations by year: atrial fibrillation ablations (top), atrial flutter ablations (middle) and atrioventricular node ablations (bottom).

The mean age of the AF (p < 0.001), AFL (p < 0.001) and AVN (p = 0.007) ablation patients increased significantly during 2012–2018 (Table 1, Table 2, Table 3). Most of the AF and AFL ablation patients were 50–69 years old (71.3 % in AF, 62.8 % in AFL). During the observation period the use of AF and AFL ablation increased especially in the age group of 70–79-year-old patients (p < 0.001 for both) but decreased among AF patients aged 40–49 years (p < 0.001) and AFL patients aged 40–59 years (p = 0.022) (Table 1, Table 2, Fig. 2). As comes to the AVN ablations, most of the patients were over 70 years old (67.3 %) (Table 3, Fig. 2). The proportion of patients undergoing AVN ablation increased particularly in the age groups of over 70-year-old patients from 57.9 % to 73.1 % (p = 0.004) whereas it decreased in the age group 60–69 years from 28.1 % to 15.5 % (p = 0.027).

The gender differences in AFL ablations attenuated over the study period, but no significant shifts in gender distributions were noted in the AF and AVN groups over time. (Table 1, Table 2, Table 3).

The mean CHA_2_DS_2_-VASc-score increased in the AF ablation group from 1.6 to 1.8 and in the AFL ablation group from 2.0 to 2.4 during the observation period (p < 0.001 in both) (Table 1, Table 2). CHA_2_DS_2_-VASc score was highest in the AVN ablation group, but the change, from 4.3 to 4.5, was not statistically significant (p = 0.310) (Table 3). Same trends were also observed in the modified CHA_2_DS_2_-VA score which does not include sex category.

Of AF ablation patients, 31.1 % underwent their first procedure within two years and 56.3 % within five years from the first AF or AFL diagnosis. (Appendix 3) The proportion of patients undergoing first AF ablation within two years increased from 23.4 % in 2012 to 32.7 % in 2018 (p = 0.003). Moreover, 48.0 % of the AFL ablation group underwent their first procedure within two years and 69.2 % within five years from diagnosis and in the AVN group these proportions were 21.6 % and 48.2 %.

A total of 986 (20.1 %) patients in the AF ablation group underwent two or more AF ablations, the mean time from the first to the second AF ablation being 1.5 years. Patients who underwent only one AF ablation were older (59.2 vs 58.5 years, p = 0.035) and had higher prevalence of diabetes (7.9 % vs. 5.9 %, p = 0.035) when compared to their peers undergoing redo ablations. Altogether 247 (9.0 %) patients in the AFL ablation group had redo AFL ablations, the mean time from the first to the second ablation being 1.3 years.

A total of 907 (11.5 %) from 7915 patients underwent both AF and AFL ablations and of them, 549 had their first AF and AFL ablations during the same procedure. Furthermore, 70 (0.9 %) patients underwent both AF and AVN ablations in separate sessions, 79 (1.0 %) patients underwent both AFL and AVN ablations and 17 (0.2 %) patients underwent all three, AF, AFL and AVN ablation procedures during the observation period. Out of 2731 AFL ablation patients, 2545 (93.2 %) underwent typical AFL ablation, 314 (11.5 %) atypical AFL ablation and 128 (4.7 %) underwent both types of AFL ablation procedures. Moreover, 199 (7.3 %) patients in AFL ablation group had AF ablation before their first-time AFL ablation and 48 (1.8 % of all AFL ablations) of these AFL procedures after AF ablation were atypical AFL ablations. Most of the patients in both typical and atypical AFL ablation groups were men (75.4 % and 65.0 %, respectively). The mean age in the typical AFL ablation group was 62.4 years and in the atypical AFL ablation group 61.2 years. There were no differences in the CHA_2_DS_2_-VASc scores (2.2 in both groups) or CHA_2_DS_2_-VA scores (1.9 in typical AFL ablation group and 1.8 in atypical AFL ablation group, p = 0.262).

**Fig. 3:**
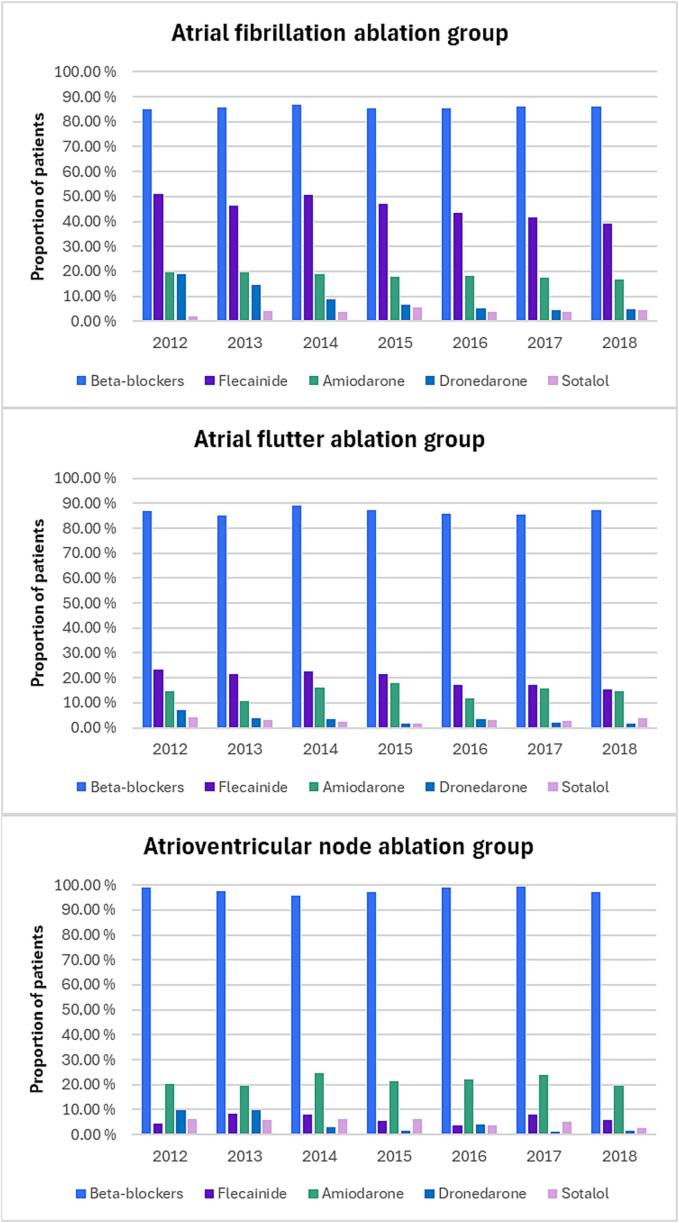
Proportion of patients that had used beta-blockers, flecainide, amiodarone, dronedarone or sotalol within a year before the first ablation.

In 2012 76.6 % of first-time AF ablation patients had used either flecainide, amiodarone, dronedarone or sotalol within a year before the ablation and the proportion decreased to 56.6 % by year 2018 (p < 0.001). (Fig. 3, Appendix 4) Also, in the AFL ablation group the use of these AADs before ablation decreased significantly from 41.3 % in 2012 to 32.1 % in 2018 (p = 0.043). In the AVN ablation group these proportions were 34.2 % and 27.3 %, but the change was not statistically significant. (p = 0.44). What comes to the differences in the use of AADs in different age groups, 62.3 % of under 60 years old patients and 67.1 % of 60 years or older patients in AF ablation group had used AADs within a year before first ablation. These proportions were 38.6 % and 33.3 % in AFL ablation group and 38.6 % and 32.1 % in AVN ablation group. Almost all patients (97.9 %) from AVN ablation group had used beta-blockers within a year before ablation, whereas these proportion were 85.8 % and 86.6 % in the AF and AFL ablation groups, respectively.

## 4 Discussion

The main findings in this nationwide retrospective cohort study were:

1. The volumes of first-time catheter ablation procedures to treat AF and AFL more than doubled through the observation period from 2012 to 2018, but the number of yearly procedures remained low when compared to the total number of patients diagnosed with AF and AFL.

2. Most of the increase in the use of AF and AFL catheter ablation occurred in 70–79-year-old patients.

3. The use of AVN ablations increased in patients over 70 years of age and decreased in 60–69-year-old patients.

4. The age and CHA_2_DS_2_-VASc as well as CHA_2_DS_2_-VA scores of patients undergoing ablation procedures increased over time.

5. AF and AFL ablations were performed predominantly to men whereas patients undergoing AVN ablations were more often women.

Our findings of the increasing use of catheter ablation to treat AF and AFL in Finland are in line with global trends. The continuously increasing procedural volumes most likely reflect the paradigm shift in the 2010 s accepting catheter ablation as the first-line treatment option in selected AF patients which is prone to increase the use of ablation in the treatment of AF and AFL. [6], [10] Also, the emergence of new ablation techniques, such as cryoballoon ablation, could have promoted the use of catheter ablation instead of antiarrhythmic drugs. We found that most of the patients in AF ablation group had used AADs prior to ablation but on the other hand the use of AADs prior to ablation decreased over time which could signal the implementation of the changes in the AF guidelines to clinical practise.

Moreover, AF prevalence increased during the observation period, due to ageing of the population and probably also due to more pragmatic AF screening. [1], [5], [13], [14] Nevertheless, the total number of yearly first-time AF, AFL and AVN ablation procedures has remained low, when compared to all patients diagnosed with AF and AFL, thus only a small proportion (0.38–0.65 %) of patients were treated with catheter ablations (Fig. 1, Appendix 2). Catheter ablation is a very resource intensive treatment method for AF and AFL, and there is constantly increasing scarcity of healthcare resources also in Finland. So, it is essential to gain more knowledge on the most cost-effective ways to treat the increasing number of patients with AF and AFL. However, with these numbers in mind, it is fair to assume that the vast majority of the AF and AFL-related healthcare costs are not associated with catheter ablations.

The age of the patients treated with catheter ablation increased during 2012–2018. Most probably this projects the ageing of the population and the increased prevalence of AF particularly in the elderly. [15], [16] The evidence supporting catheter ablation treatment in older patients is limited and remains controversial. Early rhythm-control therapy has been associated with lower risk of adverse cardiovascular outcomes also in the elderly and in patients with multiple cardiovascular comorbidities. [11], [17], [18] On the other hand, prospective randomized studies such as MANTRA-PAF and CASTLE-AF, as well as the more recent CABANA-trial reported no prognostic benefits in the elderly patients. [19], [20], [21] A recent *meta*-analysis observed that there was no difference in the success rate of AF ablation between older and younger patients, but the older patients experienced more often procedure related complications. [22] Our finding that especially 70–79-year-old patients are treated increasingly with catheter ablations signals that patients who were previously considered to be “too old” are nowadays more often treated invasively.

A great majority of AF and AFL ablation patients were men, whereas more than half of AVN ablation patients were women. Previous publications have reported that women are less likely to undergo catheter ablation procedures for AF and several possible explanations for that have been described. [23] For example, this is likely related to the higher incidence of AF among men and older age of women at the time of AF diagnosis. Moreover, previous studies have suggested that women have a higher risk of procedural complications, especially vascular complications due to smaller body size, but also gender disparities favouring men in the use of rhythm control therapies have been suggested. [24], [25], [26], [23] However, based on our current descriptive analysis we are unable to draw definitive conclusions on gender inequality in the use of invasive rhythm control strategies among patients with AF and AFL.

We found that even though the use of AVN ablation increased during 2012–2018, the increase took place predominantly in patients aged 70 years or older whereas the proportion of procedures performed in the age group of 60–69-year-old patients decreased. According to the recent *meta*-analysis the solid long-term evidence supporting AVN ablation and pacemaker implantation as a treatment strategy for AF is scarce. In previous reports, especially younger patients with reduced left ventricular function have presented with increased cardiac mortality after AVN ablation and right ventricular pacing, but also contradictory results about safety and efficacy of this treatment strategy have been published. [8], [27] In our cohort, a great majority of patients who underwent AVN ablation were from the outset without prior AF or AFL ablation. However, patients in the AVN ablation group were also significantly older, had higher CHA_2_DS_2_-VASc and CHA_2_DS_2_-VA scores and had less frequently used AADs prior to ablation in comparison to those who were treated with AF or AFL ablation. These findings suggest that most of the patients in the AVN ablation group were considered unsuitable for rhythm control treatments.

Patients who underwent redo AF or AFL ablations were younger and had lower prevalence of diabetes compared to their peers who had only one ablation. Unfortunately, we do not have data on the success rate of the ablation, but instead studied the differences in clinical characteristics between patients undergoing single ablation procedure and those scheduled for redo ablations. Thus, one cannot conclude that the success rate in younger and healthier patients is lower, but most likely these findings indicate that in case of recurrence of AF or AFL, younger and healthier patients are more likely to be scheduled for redo procedures.

Earlier studies have reported that the time from AF diagnosis to ablation predicts AF recurrences and that early ablation treatment is associated with better success. [11], [28], [29], [30], [31] In our cohort one third of the patients underwent AF ablation within two years of first AF diagnosis at the end of the observation period. However, one should bear in mind that in our study the follow-up started from the first-ever AF or AFL diagnosis recorded in the national registries rather than from the time the patient was put into the waiting list for catheter ablation. In addition, the AF or AFL diagnosis was not based only in the diagnosis established in the tertiary care but the data included also AF and AFL diagnoses from the primary care registries whereas most of the previous reports included only patients and AF diagnoses from hospital registries or from otherwise selected groups of patients, for example patients with healthcare insurance coverage. [29], [32], [33].

The major strength of this study is that the FinACAF-study cohort is a comprehensive nationwide dataset including all patients with AF and AFL and it covers all patients undergone AF, AFL and AVN catheter ablation procedures in Finland between 2012 and 2018. That provided us a unique opportunity to evaluate the procedural trends, proportion of patients provided an invasive procedure, and clinical characteristics of the patients scheduled for catheter ablation in an unselected spectrum and from all levels of healthcare system. Moreover, this data allows us to evaluate the changes in the use of different treatment modalities for AF and AFL in clinical practice over time and to understand better the total burden of different AF and AFL treatments to healthcare system. Our dataset currently provides one of the most comprehensive evaluations of the use of catheter ablation treatment in total nationwide AF and AFL population. Similar studies are needed from other countries, but trends of this study most likely describe the development of the use of catheter ablations for AF and AFL treatment in other countries as well.

On the other hand, we must acknowledge some limitations, most of which are typical and inherent in register-based retrospective cohort studies. For example, a possibility of some inaccuracies in recordings of the data, even though the registers used in this study has previously been discovered very consistent particularly in the detection of cardiovascular diseases. [34] We also lack information about some meaningful data regarding catheter ablation treatment of AF and AFL, for example information on method used for AF ablation (cryoballoon or radiofrequency ablation), the number of procedures performed by each facility or physician, AF related symptoms, AF recurrences, AF burden, body mass index, echocardiographic findings, and smoking status. Moreover, we found that 7.3 % of the patients with first-time AFL ablation had previously undergone AF ablation procedure and 1.8 % of these first-time AFL procedures after AF ablation were atypical AFL ablations. Due to registry-based nature of the data we did not have access to patients’ medical records. Thus, it is possible that some patients undergoing ablation of atypical AFL had iatrogenic atrial tachycardia or left atrial flutter secondary to the earlier AF ablation. It is important to notice that there are clinical differences between typical atrial flutter that occurs after atrial fibrillation ablation and left atrial tachycardia.

In conclusion, in this comprehensive nationwide cohort study including all AF and AFL patients in Finland between 2012–2018 we found that first-time catheter ablation procedures to treat AF and AFL more than doubled, the age of the patients’ undergoing ablations increased, and that most of the patients undergoing AF and AFL ablations were men whereas those scheduled for AVN ablation were more often women. However, the number of yearly first-time catheter ablation procedures remained low when compared to the total number of patients with AF and AFL.

## 6 Role of the funder/sponsor

The funders had no role in the design and conduct of the study; collection, management, analysis, and interpretation of the data; preparation, review, or approval of the manuscript; and decision to submit the manuscript for publication.

## 7 Registration number of clinical studies

ClinicalTrials Identifier: NCT04645537; ENCePP Identifier: EUPAS29845.

## 8 Conflict of interest disclosures

Antti Lappalainen: Research Grants: The Finnish State Research Funding, The Finnish Foundation for Cardiovascular Research, Ida Montin Foundation, Support for attending meetings: The Finnish Cardiac Society, University of Eastern Finland. Juha E.K. Hartikainen: Research grants: The Finnish Foundation for Cardiovascular Research, EU Horizon 2020, EU FP7. Advisory Board Member: BMS-Pfizer-alliance, Novo Nordisk, Amgen. Speaker: Novo Nordisk, Bayer. Konsta Teppo: Research grants from The Finnish Foundation for Cardiovascular Research, The Finnish Medical Foundation, The Finnish Foundation for Alcohol Studies and the Finnish State Research Funding. Olli Halminen: none. Aapo L. Aro: Research grants: Finnish Foundation for Cardiovascular Research, Sigrid Juselius Foundation. Speaker: Abbott, Johnson & Johnson, Sanofi, Bayer, Boehringer-Ingelheim Rasmus Siponen: none. Janne Virrankorpi: none. Annukka Marjamaa: none. Birgitta Salmela: Speaker: BMS-Pfizer alliance, Boehringer Ingelheim; Advisory board: Pfizer; Educational Grants: Medtronic, Abbott. Jukka Putaala: Speaker: Bayer, Boehringer-Ingelheim, BMS-Pfizer, Abbott; Advisory board: Portola, Novo Nordisk, Herantis Pharma; Visiting editor: Terve Media; Stock ownership: Vital Signum. Pirjo Mustonen: Consultant: Roche, BMS-Pfizer-alliance, Novartis Finland, Boehringer Ingelheim, MSD Finland, Bayer. Miika Linna: Speaker: BMS-Pfizer-alliance, Bayer, Boehringer-Ingelheim. Jari Haukka: Research grants: The Finnish Foundation for Cardiovascular Research, and EU Horizon 2020, EU FP7. Advisory Board Member: BMS-Pfizer-alliance, Novo Nordisk, Amgen. Speaker: Cardiome, Bayer. K.E. Juhani Airaksinen: Research grants from the Finnish Foundation for Cardiovascular Research and Clinical Research Fund of Turku University Hospital, Turku, Finland. Lectures for Astra Zeneca, Bayer, Boehringer Ingelheim, Mika Lehto: Consultant: BMS-Pfizer-alliance, Bayer, Boehringer-Ingelheim, and MSD; Speaker: BMS-Pfizer- alliance, Bayer, Boehringer Ingelheim, MSD, Terve Media and Orion Pharma. Research grants: Aarne Koskelo Foundation, Yrjö Jahnsson Foundation, The Finnish Foundation for Cardiovascular Research, and Helsinki and Uusimaa Hospital District research fund.

## CRediT authorship contribution statement

**Antti Lappalainen:** Writing – original draft, Visualization, Methodology, Investigation, Formal analysis, Data curation, Conceptualization. **Juha E.K. Hartikainen:** Writing – review & editing, Supervision, Methodology, Conceptualization. **Konsta Teppo:** Writing – review & editing, Methodology, Formal analysis, Conceptualization. **Olli Halminen:** Writing – review & editing, Methodology, Investigation, Formal analysis, Data curation, Conceptualization. **Aapo L. Aro:** Writing – review & editing, Supervision, Methodology, Conceptualization. **Rasmus Siponen:** Writing – review & editing, Conceptualization. **Janne Virrankorpi:** Writing – review & editing, Conceptualization. **Annukka Marjamaa:** Writing – review & editing, Supervision, Methodology, Conceptualization. **Birgitta Salmela:** Writing – review & editing, Supervision, Methodology, Conceptualization. **Jukka Putaala:** Writing – review & editing, Supervision, Methodology, Conceptualization. **Pirjo Mustonen:** Writing – review & editing, Supervision, Methodology, Conceptualization. **Miika Linna:** Writing – review & editing, Supervision, Methodology, Conceptualization. **Jari Haukka:** Writing – review & editing, Supervision, Methodology, Data curation, Conceptualization. **K.E. Juhani Airaksinen:** Writing – review & editing, Supervision, Methodology, Conceptualization. **Mika Lehto:** Writing – review & editing, Supervision, Project administration, Methodology, Funding acquisition, Conceptualization.

## Funding

This work was supported by 10.13039/100014714The Finnish State Research Funding, The Finnish Foundation for Cardiovascular research, Ida Montin Foundation, Aarne Koskelo Foundation, Yrjö Jahnsson Foundation and Helsinki and Uusimaa Hospital District research fund (TYH2019309 and TYH2023319).

## Declaration of competing interest

The authors declare that they have no known competing financial interests or personal relationships that could have appeared to influence the work reported in this paper.
