# Supplementary material for: Temporal trends of catheter ablation procedures in patients with atrial fibrillation and atrial flutter: A nationwide cohort study
Source: Int J Cardiol Heart Vasc. 2024 Oct 23;55:101541. doi: 10.1016/j.ijcha.2024.101541 (PMC11539521; doi:10.1016/j.ijcha.2024.101541)
Supplement: Supplementary Data 1 [file mmc1.docx]

**Appendix 1.** Flow-chart of the patient selection process.


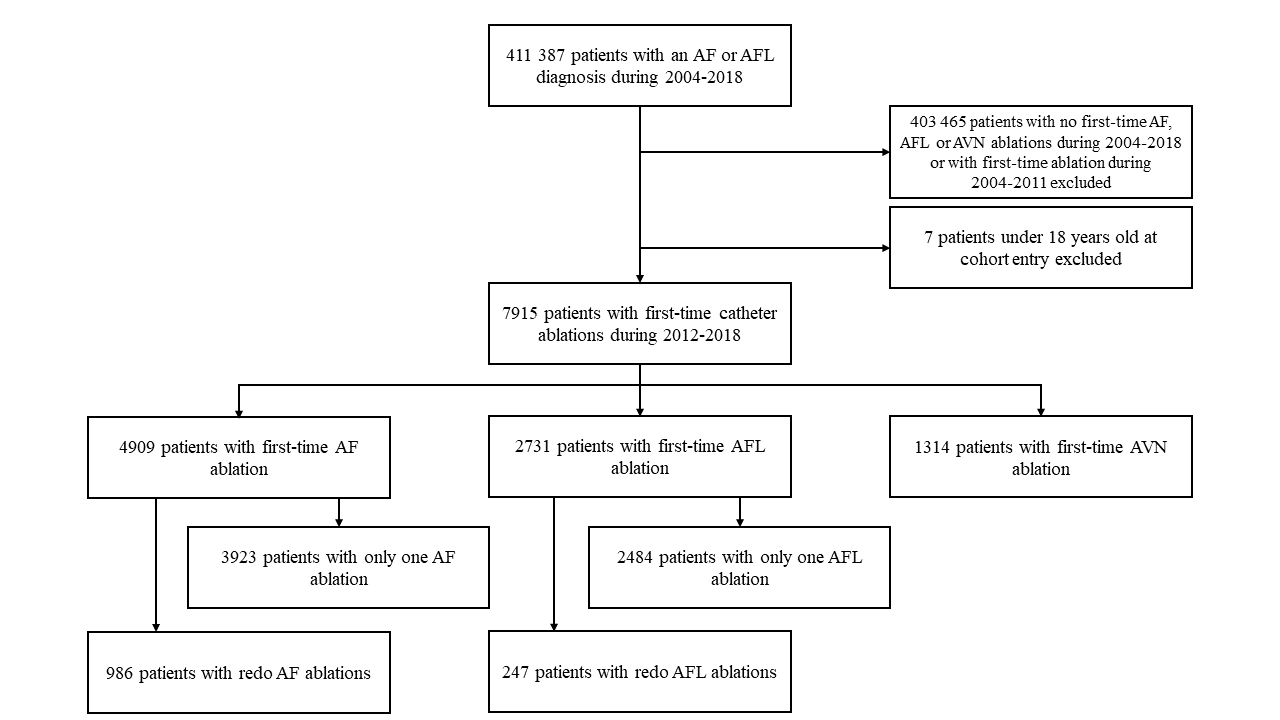


Abbreviations: AF, atrial fibrillation. AFL, atrial flutter. AVN, atrioventricular node.

**Appendix 2.** Prevalence of atrial fibrillation and atrial flutter and number of first-time catheter ablations by year.

|  | **2012** | **2013** | **2014** | **2015** | **2016** | **2017** | **2018** | **Total** |
| --- | --- | --- | --- | --- | --- | --- | --- | --- |
| Total number of prevalent patients | 185 057 | 197 536 | 207 998 | 217 065 | 226 637 | 235 386 | 243 802 | 335 743 |
| Atrial fibrillation ablations | 457 (0.25) | 570 (0.29) | 594 (0.29) | 691 (0.32) | 788 (0.35) | 875 (0.37) | 934 (0.38) | 4909 (1.46) |
| Atrial flutter ablations | 223 (0.12) | 232 (0.12) | 315 (0.15) | 380 (0.18) | 481 (0.21) | 547 (0.23) | 553 (0.23) | 2731 (0.81) |
| Atrioventricular node ablations | 114 (0.06) | 122 (0.06) | 164 (0.08) | 197 (0.09) | 223 (0.10) | 256 (0.11) | 238 (0.10) | 1314 (0.39) |
| All first-time ablations | 794 (0.42) | 924 (0.47) | 1073 (0.52) | 1268 (0.58) | 1492 (0.66) | 1678 (0.71) | 1725 (0.71) | 8954 (2.67) |
| Number of all ablation patients | 700 (0.38) | 792 (0.40) | 905 (0.44) | 1108 (0.51) | 1326 (0.59) | 1489 (0.63) | 1595 (0.65) | 7915 (2.36) |

Values denote n (%).

**Appendix 3.** Catheter ablations within two and five years from cohort entry.

|  | **2012** | **2013** | **2014** | **2015** | **2016** | **2017** | **2018** | **Total** | **p-value** |
| --- | --- | --- | --- | --- | --- | --- | --- | --- | --- |
| **Atrial fibrillation** | **457** | **570** | **594** | **691** | **788** | **875** | **934** | **4909** |  |
| <2 Years | 107 (23.4) | 165 (28.9) | 178 (30.0) | 219 (31.7) | 272 (34.5) | 282 (32.2) | 305 (32.7) | 1528 (31.1) | **0.003** |
| <5 years | 251 (44.0) | 329 (57.7) | 341 (57.4) | 396 (57.3) | 464 (58.9) | 472 (53.9) | 511 (54.7) | 2764 (56.3) | 0.372 |
| **Atrial flutter** | **223** | **232** | **315** | **380** | **481** | **547** | **553** | **2731** |  |
| <2 years | 106 (47.5) | 120 (51.7) | 146 (46.3) | 183 (48.2) | 241 (50.1) | 271 (49.5) | 245 (44.3) | 1312 (48.0) | 0.417 |
| <5 years | 170 (76.2) | 168 (72.4) | 212 (67.3) | 270 (71.1) | 346 (71.9) | 380 (69.5) | 345 (62.4) | 1891 (69.2) | **0.002** |
| **Atrioventricular node** | **114** | **122** | **164** | **197** | **223** | **256** | **238** | **1314** |  |
| <2 years | 29 (25.4) | 23 (18.9) | 40 (24.4) | 47 (23.9) | 46 (20.6) | 59 (23.0) | 40 (16.8) | 284 (21.6) | 0.365 |
| <5 years | 61 (53.5) | 55 (45.1) | 75 (45.7) | 95 (48.2) | 103 (46.2) | 139 (54.3) | 105 (44.1) | 633 (48.2) | 0.244 |

Values denote n (%).

**Appendix 4.** Use of any antiarrhythmic drug (flecainide, amiodarone, dronedarone, sotalol) within a year before first catheter ablation.

|  | **2012** | **2013** | **2014** | **2015** | **2016** | **2017** | **2018** | **Total** | **p-value** |
| --- | --- | --- | --- | --- | --- | --- | --- | --- | --- |
| **AF ablation** | 350 (76.6) | 406 (71.2) | 417 (70.2) | 467 (67.6) | 490 (62.3) | 520 (59.4) | 528 (56.6) | 3178 (64.7) | **<0.001** |
| **AFL ablation** | 92 (41.3) | 84 (36.2) | 124 (39.4) | 146 (38.4) | 152 (31.6) | 188 (34.5) | 177 (32.1) | 936 (35.3) | **0.043** |
| **AVN ablation** | 39 (34.2) | 46 (37.7) | 60 (36.6) | 65 (33.0) | 71 (31.8) | 84 (32.8) | 65 (27.3) | 430 (32.7) | 0.44 |

Values denote n (%). Abbreviations: AF, atrial fibrillation. AFL, atrial flutter. AVN, atrioventricular node.
